# Global Fields and Migration Regimes: Citizenship by Investment

**DOI:** 10.1111/1468-4446.70011

**Published:** 2025-07-24

**Authors:** Kristin Surak

**Affiliations:** ^1^ Department of Sociology London School of Economics London UK

**Keywords:** Bourdieu, citizenship, fields, mobility, global fields, migration

## Abstract

In the past decade, scholars of international migration have made remarkable strides in unpacking the complex infrastructures that channel cross‐border mobility by investigating the operation of profit‐oriented migration industries and the regulatory tussles of multilevel migration governance. However, little work has combined the insights of both to reveal how they interact to facilitate or inhibit the growth of particular migration regimes. This article integrates the two strands by reconceptualizing them as part of the same global field, which offers resources for exploring how the struggle for profit intersects with competitions over regulatory capital. It clarifies these dynamics through a case study of the sale of citizenship to wealthy individuals. Focusing first on the involvement of regulatory capital in the competition around economic capital, it shows how and with what outcomes countries and firms cooperate or compete in the system, leading to program resilience or risks. Then turning to the involvement of economic capital in competitions leveraging regulatory capital, it reveals how global powers can influence the citizenship policies of other countries and how third powers dominate in different ways, impacting program growth and profitability. The upshot offers greater traction for examining the limits of state sovereignty and reveals how migration regimes are produced within uneven global playing fields structured by fundamental doxa.

## 1 Introduction

In the past decade, studies of international migration have made remarkable strides in unpacking the complex infrastructures that condition cross‐border mobility. Scholars of migration industries have revealed how meso‐level businesses and agents facilitate or inhibit cross‐border movement for profit and in interaction with states (Hernández‐León 2008; Gammeltoft‐Hansen and Nyberg Sørensen 2013; Xiang and Lindquist 2014; Cranston et al. 2018). At the same, the literature on multilevel migration governance has drawn attention to how the regulations channeling movements are produced by not only states, but also supra‐national institutions, other national‐level actors, and local‐level authorities (Zincone and Caponio 2006; Panizzon and Van Riemsdijk 2019; Caponio and Jones‐Correa 2018; Scholten and Penninx 2016; Triandafyllidou 2022). Despite these advances, we know little about exact ways that the for‐profit dynamics around migration industries intersect with the regulatory scrambles of multiple levels of migration governance to facilitate or limit mobility.

This article unites these arenas by reconceptualizing them as part of the same global field to offer a more precise account of how the dynamics of profit and control interact to enhance or limit mobility regimes. It begins with an overview of the literature on migration industries and multilevel migration governance literature and proposes that a global field approach can be used to integrate the two. It then offers an overview of global field analysis as a most recent iteration of a longer history of field approaches heavily influenced by the work of Bourdieu. Drawing on new developments in the study of global fields, it specifies how more traditional approaches need to be adjusted when applying them to transnational phenomena and identifies the analytic benefits gained when studying fuzzy global fields. In doing so, it advances the concept of “regulatory capital” as a field‐specific capital that opens up for analysis the state's variable authority to regulate.

To examine how migration regimes can be analyzed as a global field, the article offers a case study of the sale of citizenship to wealthy individuals. It investigates how both the migration industry and public sector actors at multiple levels of migration governance cooperate and compete over regulatory and economic capital, continuously transforming the migration regime. By focusing on the dynamic competitions and collaborations within the field, the analysis reveals when and how the struggle over regulatory power intersects with competition over profits to facilitate or inhibit growth, and how powerful actors leverage symbolic capital to reinforce their position while also dominating differently. Attending to the dynamics of capital accumulation and conversion exposes how and why external actors can leverage regulatory power to gain influence over another state's citizenship policies, even without the legal backing to do so. It also shows how symbolic capital structures the field and its power dynamics through fundamental misrecognitions. The article concludes by suggesting other migration regimes that might be fruitfully investigated with such an integrative approach.

## 2 Migration Industries and Multilevel Migration Governance

International migration depends on infrastructures that enable people to move across borders. Since the early 2000s, a rich body of work on migration industries has revealed how an array of meso‐level actors, including specialized firms, sub‐contractors, and informal businesses, facilitate or inhibit cross‐border mobility (see, among others, Hernández‐León 2008; Gammeltoft‐Hansen and Nyberg Sørensen 2013; Xiang and Lindquist 2014; Cranston et al. 2018). An important subsection of this scholarship investigates how the private sector interacts with states to channel migration flows. Sometimes migration industries help people subvert or circumvent migration regulations (Spaan and van Naerssen 2018; Hernández‐León 2013). Frequently, though, states partner with firms to implement policies (Surak 2013, 2018). Businesses, too, may lobby states to expand their role in—and profit‐making off—migration policy implementation as they operate at the interface of profit and regulation (Menz 2009; Kalm 2023). However, the public sector does not hold all the cards. When migration businesses assume regulatory roles, they may be able to influence political decision‐making in their own interest (Kalm 2023; Axelsson and Pettersson 2021). Their guidance often occurs behind closed doors through formal lobbying, feedback sessions, or personal connections (Freeman 1995; Menz 2009; Axelsson and Pettersson 2021), though some intermediaries move debate and pressure tactics into the public sphere (Strathem and Geddes 2006). For states, such partnerships may fill a resource gap, simplify complexity, or supply targeted expertise (Nehring and Yang 2021; López‐Sala and Godenau 2022; Sánchez‐Barrueco 2018). They may also shift liabilities away and shield the state from responsibility or criticism (Guiraudon and Lahav 2000; Menz 2013; Surak 2018).

However, states engage with not only migration industries, but also other governance bodies when managing migration—a key point rarely theorized within the migration industries literature. Over the past decade, studies of multilevel migration management have challenged state‐centric approaches to dissect the involvement of subnational, supranational, and lateral public‐sector actors in migration policy development and implementation (see, among others, Zincone and Caponio 2006; Panizzon and Van Riemsdijk 2019; Caponio and Jones‐Correa 2018; Scholten and Penninx 2016; Triandafyllidou 2022). Some scholars focus on the cooperative relationships between the public‐sector actors at different levels of governance (Scholten and Penninx 2016; Moreno‐Lax 2018). Others draw out the ways in which conflict or uncoupling between levels is common and coordination rare (Spencer 2018; Niemann and Zaun 2018; Newton 2018). Much of this research has focused on the regulatory structures in vertically nested settings, such as the EU or federated systems in North America. However, some work has begun to explore at how third states or other regional organizations may become involved in migration management (Bisong 2018, see also Niemann and Zaun 2023). These external connections are more likely when actors link migration to other important issues to gain leverage (Hampshire 2015; Fakhoury 2019; Adamson and Tsourapas 2019). The upshot is a better understanding of role that governance actors beyond the state play in migration management.

At present, studies of migration industries and of multilevel migration management remain disconnected from each other. Analyses typically treat the two domains as independent despite some promising indica of confluence. Early work on multilevel migration management, for example, noted an “outward” shift of governance to private actors (e.g. Lahav 2000), yet this angle has remained largely unexplored within multilevel governance analyses (Caponio and Jones‐Correa 2018). When non‐government actors are incorporated, they become subsumed into the governance framework: that is, they are investigated only in so far as they, too, are governance actors involved in the negotiated governance order (e.g. Spencer 2018). The governance framing, however, leaves little opening to consider how other aspects—such as the monetary logic of migration industry actors—can impact the wider system. We know from the migration industry literature that the interface between public and private sectors can influence outcomes, but we have yet flesh out how they may also reverberate into more complex governance arrangements among hierarchies of political actors.

I suggest these two arenas can be brought together by conceptualizing them as part of the same global field. Global field analysis offer analytic tools for disaggregating the specific stakes, dynamics, and interactions concerning profit as well as regulation at multiple levels that condition migration regimes. It supplies leverage for identifying the ways in which—and the effects that occur when—the regulatory demands and profit imperatives intersect. It also furnishes tools for unmasking the dynamics of symbolic power and the doxic principles that inform a field's operational assumptions and limits. Finally, it decenters the state by casting it as housing one set of actors among many involved in sustaining migration regimes.

## 3 Global Field Analysis

Field analysis is an analytic approach whose sociological variant developed from of the work of several scholars but has been heavily influenced by Pierre Bourdieu (1984), (1993), (1996b); see Martin 2003). It takes as its starting point the existence of unequal actors in different positions and in possession different volumes of resources and power, or “capital” (Bourdieu and Wacquant 1992; see also Martin 2003, Go and Krause 2016, DiMaggio and Powell 1983). The approach focuses analysis on the power dynamics of how actors struggle to accumulate capital by leveraging and converting their available resources and power. Field analysis also draws attention to the normative dimension of the fundamental, or “doxic,” assumptions that define the space of play: capital competition and conversions can also challenge and potentially rework the doxa that structure a field (Bourdieu and Wacquant 1992).

Applying the tools of field analysis to understand migration regimes means examining social spaces that are not bounded by countries. This requires loosening some of the expectations built into Bourdieu's foundational work, particularly regarding the state. Many of the domains Bourdieu analyzed—the bureaucratic field, the art field, the legal field, the academic field—were deeply conditioned by the centralization of the modern state and they, too, contributed to wider state‐building efforts, a situation that recurisvely informed his theorization and contributed to a relatively bounded conceptualization of fields. As such, it may be misdirected to transpose a traditional field analysis into an international arena whole cloth (Vauchez 2011). As subsequent researchers have shown, a modified field approach that remains open to fuzziness around fields—that is, to recognizing that they are not clearly and distinctly bounded—offers a more generative framework for analyzing global fields (Steinmetz 2008; Vauchez 2011). Indeed, such flexibility is important for enabling field analysis to develop as an analytic approach rather than freezing it as a static formal system (see Martin 2003).

Over the past two decades, several scholars have done just that: they have expanded field analysis to address transnational or global phenomena (Steinmetz 2008; Vauchez 2011; Go and Krause 2016; Schmidt‐Wellenburg and Bernhard 2020; Buchholz 2016; Fourcade 2006). A global field analysis requires, at minimum, positioned and positioning actors that react to one another and compete for capital (Go and Krause 2016, 17). To this can be added agreement over the stakes of competition—the symbolic capital of the field—and control over access to the field (Steinmetz 2018). Stretching beyond the state to examine global fields draws attention to the ways that boundaries of the nation‐state themself can be part of the field's internal structure and even its stakes (see Go and Krause 2016). It also turns the spotlight toward transborder actors and interactions that structure their operation (Vauchez and France 2020). However—and importantly—global field approaches have stressed that the fuzziness around a field and fuzzy overlaps between fields are to be expected (Steinmetz 2008; Vauchez 2011). As such, many are not the familiar self‐contained realms that Bourdieu analyzed: that is, they are often “weak fields” (Vauchez 2011).

Some may argue that weak fields are not really fields and therefore little benefit is to be gained by shoe‐horning phenomena into them. The real question, however, is whether the framework offers resources for illuminating dynamics otherwise occluded. Such an approach characterizes Julian Go's theorization of the global field, or the space in which states and other actors, including international organizations and corporations, compete for multiple species of capital (Go 2008, 206–7).^1^ Its varied topography encompasses subsets of competitions, such as the imperial competitions involving “security capital” that he empirically analyses. Go's global field approach offers several advantages for capturing the dynamics of transnational power politics.^2^ Fundamentally, it operates as a “provisional theory” that supplies an orientational guide for analysis that can alert scholars to mechanisms or dynamics that otherwise remain hidden (Bourdieu and Wacquant 1992; Martin 2003). It draws attention to a multidimensional topography in which forms of capital are wielded in competition by several kinds of actors (Go 2008; see also Swartz 1997). It sharpens analysis of the “rules of the game” that are otherwise taken for granted (Go 2008). And it sensitizes analysts to the strategies that actors in dominant and subordinate positions employ in the pursuit of capital, as well as the conversion of capital into different forms (Go and Krause 2016).

I suggest that this kind of approach can be applied to the dynamic action structuring a migration regime at the intersection of migration industries and multilevel migration management. For studies of international migration and mobility, at least three benefits are gained by adopting global fields as an analytic framework. The first concerns problematizing the power of the state. Even if migration is transnational by definition, prominent strands within the literature reflect the traditional assumption that states hold a monopoly over the legitimate control of their territorial borders.^3^ They may transfer or parcel out this sovereign power by “deputizing” other actors to carry out related functions—and often do—yet they remain the seat of power (FitzGerald 2019; see also Cooley and Spruyt 2009). Studies also typically regard the state as having acquired a monopoly on the legitimate control of membership, (e.g. Torpey 1999; Brubaker 1992; Hollifield 2006). However, a global fields approach opens up possibilities to capture and analyze challenges to these monopolies. Attending to the dynamics of capital accumulation and conversion can reveal how and why, for example, external actors can gain influence over a state's citizenship policies, even without the formal legal backing to do so. That is, global fields enable state monopolies to move from being part of the *explanans* to being also a part of the *explanandum*.

Second, it enables the integration of two key dimensions that structure mobility: profit and regulation. Traditionally, the literature on migration industries focused on the impact of profit on the infrastructures that channel migration while the multilevel migration management literature addressed governance. A global field approach enables these to be integrated. It draws attention multiple forms of capital competition and opens analysis of how vectors normally treated separately—such for‐profit action or regulatory action—intersect and influence each other (see Go 2008). With global fields, it becomes possible to dissect the complex dynamics and competitions not only within the migration industry or within multilevel migration management, but also between them. Focusing on conversions of capital, as well as struggles over its value, recognition, fungibility, and volume enables analysis of how, why, and when competition between multiple public‐sector actors concerned with regulation impacts the competition for profit that involves both governments and businesses—and to what degree the reverse occurs as well.

To do so, this article suggests the utility of “regulatory capital” as a field‐specific capital for analyzing the competition around the authority and capacity to regulate. Fundamentally, regulatory capital refers to the power and resources to establish and implement policy. It is generated by legal frameworks, including foundational documents like constitutions or treaties, as well as institutional infrastructures (see Weber 1978). In many ways, it contains much in common with Michael Mann's concept of “infrastructural power” held by states (Mann 1984). However, it extends beyond Mann's conceptualization to incorporate not merely the internal capacity to regulate but also the authority to regulate. Taking authority as variable opens avenues for investigating the ways that states do not have an inherent monopoly over regulatory power as a sovereign entity—a point often occluded by the operation of symbolic capital which projects the regulatory authority of states as given. Crucially, not all states are equally able to act as a “central bank of symbolic capital” (cf. Bourdieu 1997, 240)—particularly in global fields. Rather, some have greater stores that enable them to violate the sovereignty of other states in ways deemed legitimate. Indeed, such acts of domination, exercising symbolic power, can even strengthen the legitimacy of a global field.

Exposing the variability of regulatory authority also opens space for examining how multiple regulatory actors with differing governance capacities and purviews interact in the creation and implementation of policy, as recognized by the literature on multilevel migration management. States, with variable capital reserves, may operate in the same regulatory space as regional entities, supranational organizations, third‐sector actors, and the private sector (Guiraudon and Lahav 2000). The regulation of border control, for example, may see states deputizing the private sector and NGOs to carry out elements of enforcement and delegating regulatory authority to supranational organizations. At the same time, a global fields approach moves beyond the multilevel governance framework and its focus on complex networks (see Caponio and Jones‐Correa 2018) to provides a framework for analyzing changes in regulatory power within a competitive field of multiple governance actors with varied positionings and position‐takings. This may also involve capital conversions, as when, for example, states appoint private sector organizations to run labor migration recruitment programs, transferring regulatory functions onto a separate set of actors that subsequently leverages them for profit (see Surak 2018). Actors may also deploy their regulatory capital to generate economic capital, as for example when states establish citizenship by investment schemes to gather funds.^4^

Third, a global field approach supplies leverage for unpacking the operation of symbolic power that undergirds the fundamental assumptions, or *doxa*, that organize fields but are generally taken for granted. Competition within a field is possible only because actors have, upon entry, tacitly agreed to the fundamental rules of the game that enable play in the first place. Accepting these doxa and the wider workings of symbolic power generates legitimacy within the field and the collective “misrecognition” of the forms domination that produce the field while appearing to be self‐evident (Bourdieu 1996a, 166–169). Analysis of how dominant powers leverage symbolic capital to advance their own interests also sheds light on the arbitrariness of *doxa* that are usually taken for granted.

## 4 Inside the Field in Motion: A Case Study of Citizenship by Investment

**FIGURE 1:**
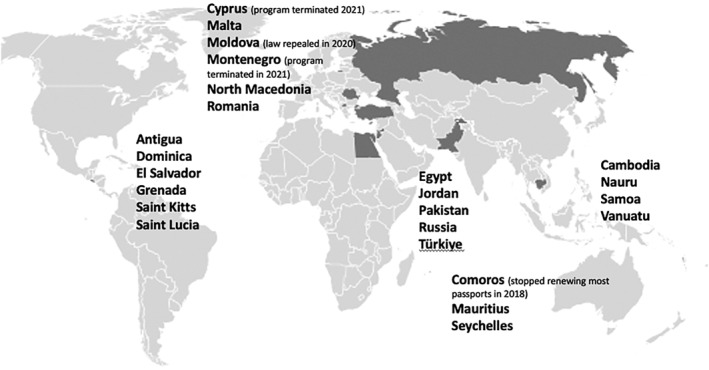
Countries with CBI legal provisions (2015–2024).

How this works in practice can be illustrated through a case study of the ways that the competition over regulatory capital and economic capital intersect in the transnational market around the sale of citizenship. Citizenship by investment programs are government‐run schemes that provide a straight‐forward, formalized pathway to naturalization based on an investment in or donation to a country. Official policies set out the investment or donation amounts, as well as the investment types, such as real estate or government bonds. Costs run typically between $100,000 to $1 million, in addition to fees, with rates often higher for each family member included. Around 20 countries have legal provisions enabling citizenship by investment (Figure 1), but less than a dozen have active programs that regularly naturalize at least 100 people annually. Approximately 50,000 individuals naturalize through the schemes each year, a figure that includes both the individual who make the investment or donation and any additional family members included on the application. Most are from the non‐West, with China, the Middle East, and Russia the traditional demand centers, although United States (US) citizens have been showing increasing interest (Surak 2023).

At the heart of these competitions is citizenship, which is undergirded by two doxic principles: the first concerns sovereignty and state membership, and the second identity and national membership. It is taken for granted that citizenship is a sovereign prerogative and only independent nation‐states can bestow it. Over the course of the nineteenth and twentieth centuries, nation‐states acquired a monopoly on the legitimate control of membership from competing power sources, and they exercise this control through citizenship regimes (Torpey 1999; Brubaker 1992; Hollifield 2006). Dependent territories like Anguilla, supra‐state associations like the European Union (EU), or sub‐state divisions like Amsterdam cannot issue their own citizenship, though some such entities were able to do so in the past (see Prak 2023).^5^ The inclusion of some within the citizenry means, simultaneously, the exclusion of others from it, and such restrictions are deemed legitimate and necessary for collective self‐determination and sovereignty to function (see Achiume 2019). With a monopoly on citizenship attribution, the state must also stand behind citizenship for it to be valid. Withdrawal of recognition renders a population stateless, as seen for example, in the case of the Rohingya (see Parashar and Alam 2018).

Although citizenship is fundamentally a legal status linking individuals to a state, a second doxic principle connects citizenship to membership in a nation (Brubaker 1992). A nation is an “imagined political community…imagined as both inherently limited and sovereign” (Anderson 2006, 6). The attribution of citizenship at birth, in most cases, connects the individual to the nation through bloodlines—even, arguably, in *jus soli* systems (Kochenov 2019). After birth, the acquisition of citizenship through naturalization is typically premised on indicating some degree of national membership. Citizenship tests, for example, ostensibly serve as indications of a person's integration into a wider national society (Etzioni 2007). A more common tool, however, is the immigrant‐into‐citizen pathway whereby the years spent in a country are regarded as evidence of integration into the national fold and therefore a legitimate basis of naturalization. For those outside a state's border, membership in a national diaspora can serve in a similar role (Joppke 2003). It is this secondary connection to the nation that sacralizes citizenship and attaches it to identity.

At stake within the field is the determination of and influence over who gets to be a citizen via naturalization and the derivation of profit or revenue from it. Within the space of competition, the most powerful actors are those in the public and private sectors.^6^ Indispensable are the countries that offer citizenship by investment programs (shortened to “CBI countries” for concision). Traditionally, they have been microstates, or countries with populations of less than 1 million. More recently, however, larger countries, including Egypt, Jordan, and Turkey have launched operational programs. Of the approximately twenty countries with CBI legal provisions, only around half naturalize significant numbers of investor citizens on a regular basis (Surak 2023). In addition, third powers, including the United States (involving mainly its Department of State and Department of Treasury sub‐branches) and the European Union (involving mainly its European Parliament and European Commission sub‐branches), have maintained active stances within the CBI field. Other actors may have some leverage that could be employed in the field but they remain largely on the sidelines. Canada and the UK have occasionally taken positions by revoking visa‐free access but are not regularly engaged in the field. International organizations like the OECD and IMF have some influence over countries granting citizenship by investment in the form of loan stipulations or blacklists, but they have only occasionally issued statements on the topic and have not been key actors in the regulatory space. Other global powers like China, as well as countries of origin of naturalizers, have not taken significant positionings.

The key private sector actors involved are those of the citizenship industry. The most powerful firms within it are “dominant consultancies” or businesses that specialize in investment migration offerings and maintain offices in several countries and sometimes several hundred employees. They may have government contracts for running aspects of programs, as well as government advisory units that carry out consulting activities and lobby countries to start schemes. These firms can also have sub‐branches or partnerships that offer additional services to deal with financing, moving money across borders, investment opportunities, real estate projects, and “aftercare,” such as subsequent paperwork. Other large players include the private wealth management arms of Big Four accountancies and international banks, as well as major international law firms. These multinational companies offer citizenship by investment options to clients and may be involved in government consultations about programs, but their main business is typically not investment migration. Mid‐sized firms also operate in this space. They may have offices in more than one country, but they are less likely to be involved in government lobbying or consultations. Small‐sized firms, with perhaps only one office, are also active players, often feeding clients on to larger firms, or submitting applications for them in CBI country itself.^7^

### 4.1 Regulatory Capital in the Competitions Around Economic Capital

**FIGURE 2:**
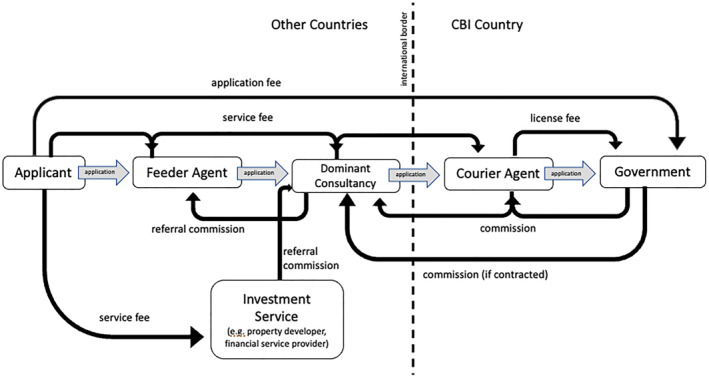
Extended supply chain example.

The generation of economic capital amassed by particular actors is the raison d’être for citizenship by investment programs. Money typically moves from investor to country along an extended supply chain that connects feeder agents who collect clients and pass them on to bigger consultancies, who then connect to courier agents in the CBI country for application submission. Commissions and fees keep everything moving along this transnational chain of business connections (Figure 2). The overall volume of economic capital available is a function of demand and supply, and dominant consultancies in the private sector drive the increase of both. Developing demand is straightforward. Usually, an experienced business moves to the target area and partners with local law firms or wealth managers that have an established a client base. It then carries out a marketing campaign to convince locals that the “citizenship product” is legitimate. If all goes well, pioneer companies complain of market saturation within a few years.

Creating supply is more challenging as it depends on leveraging one form of capital (regulatory) to expand the volume of another (economic). Service providers use the promise of economic rewards to persuade governments to deploy their regulatory capital to establish programs. Typically, a dominant consultancy will form partnerships with local law firms and related businesses in the selected country and dispatch a dedicated government advisory unit to lobby politicians. Effectively, they leverage potential economic returns to gain sway over regulatory capacity. Ready targets are states that have existent legal provisions to facilitate naturalization for exceptional circumstances since they require only policy development—rather than passing new laws—to create a program. A ready‐made legal basis is useful for it can be difficult to persuade politicians and especially local populations to support “selling citizenship,” as the doxic understanding of citizenship that attaches it to the nation means that the legal status is often regarded in highly symbolic and even sacred terms. This renders many resistant to its brute marketization, at least initially. Unsurprisingly, most programs to date have been set up in smaller, economically limited countries or by authoritarian regimes. If the lobbying tactics are successful, the result is an addition to the range of “products” that a migration agent can sell. However, the most desirable options are for countries with extensive visa‐free travel agreements or other extra‐territorial benefits like advantageous tax agreements with other states. Countries offering more substantial external legal benefits can sell their citizenship at higher rates, but because these legal benefits include those secured via treaty with other states, they can supply external countries with a lever of influence as well.

Regulatory power—and its limits—structures the field of economic competition as country borders provide profit and protection for the extended supply chain. In the first instance, state regulatory power creates differences between countries that generate information asymmetries which are rallied to create economic capital. Applicants are rarely familiar with investment opportunities in small countries they have never visited and many are willing to pay a premium to avoid the hassle of paperwork. The same differences can make it challenging for agents to have a hand in all parts of the global market, and most find it more feasible or profitable to carve out a niche. An agent may have the know‐how to submit a solid application for Saint Lucia, but not for Egypt and subcontract another firm to handle clients interested in the latter. Governments, too, are beholden to information asymmetries. Those hoping to increase their numbers may not have the knowledge or resources to recruit applications from other parts of the world and will offer commissions to agents for each approved file they bring through, sometimes increasing the premium for bulk submissions. If this reduces the amount of revenue the government acquires from each applicant, the overall intake may still be greater if approval numbers increase.

Secondarily, however, borders protect the extended supply chain from regulatory incursions when firms bend or break rules for profit. Service providers may employ illegitimate strategies to enhance their earnings, including false advertisements, illegal financing, and other dodges. A CBI government may be able to punish agents who violate the rules within its territory, if there is political will, but it has far fewer possibilities to penalize infractions that occur outside its borders. Yet states have made attempts. Many governments require applications to be submitted by locally licensed agents, which gives the state the possibility to revoke licenses when bad practices come to light. Name‐and‐shame blacklists are another strategy. Still there is little a government can do if an individual operating a firm abroad that gets banned simply sets up a new one, perhaps protected by a shell company. The geographic limits that define state sovereignty in the first instance also create barriers to state regulation and facilitate opportunities for the private sector to “play in the gray” (see Hoang 2022).

The competition for economic capital in this system is the most dynamic at the interface between the CBI governments and the private sector as states expending regulatory power create possibilities for the accumulation of economic capital. In some cases, the capital conversions strengthen the market and facilitate the field's resilience and expansion. The interface between the public and private sectors is *symbiotic* when cooperation enhances the capacity for both actors to accumulate economic capital, increasing its overall volume while sustaining the legitimacy of the field. Private actors may serve as a gatekeeper to the global market, including access to demand in distant places, and have specialized knowledge about the market's operation that is unknown to frequently country‐bound bureaucrats. Turkey, for example, did not see largescale uptake of its program for the first two years until it held consultation sessions with the private sector and assembled advice on how to increase its market share. It subsequently dropped its minimum investment amount and saw application numbers soar. Migration agents who participate in such sessions will transform their government connections into a selling point to attract clients. In these cases, the interests of the profit‐driven private sector and revenue‐hungry country economies are in alignment, leading to a possible increase in both profits and revenue.

The private sector may also *substitute* for absent state regulatory capacity and thereby facilitate the field's resilience and growth. For example, thorough background checks on applicants are a common challenge for countries with programs, which often lack the infrastructural capacity to assess the veracity of applications presenting lives and businesses in other parts of the world. Many instead appoint professional due diligence firms with a wide international network of investigators to carry out “boots on the ground” assessments of the applicants. The outsourcing substitutes private sector capacity for public sector infrastructural weakness in a way that improves vetting and diminishes the risk that criminals or terrorists will be approved (on its limits, see Surak 2023). Third powers like the OECD and US have generally praised such partnerships as good practice in the field, which can result in a more resilient program that can attract more revenue in the long term.

Regulatory capital can also be converted into profit for specific actors in ways that undermine a field's overall legitimacy and growth potential. The relationship between the public and private may turn into one of *collusion* if firms and officials team up to facilitate kickbacks or payoffs for the personal enrichment of particular politicians or public servants (Surak 2023, 2024). The most spectacular example to date is found in the Cypriot case, though it is not the only one. In 2020, Al Jazeera exposed high‐level government officials accepting kickbacks to enable naturalizations that circumvented the standard application procedures, which led to the suspension and eventual closure of the CBI program. Collusion may generate increased profits for the private sector and the personal wealth of public officials, but it can come at the expense of revenue productively enhancing the economy or of the program itself.

At the extreme, the private sector may end up *commandeering* the state, blurring the boundary between the private and public almost completely (see also Surak 2013). Vanuatu and Comoros offer examples in which service providers have arrogated state power to sell citizenship without the awareness of the ministries typically involved in naturalization, resulting in cases of such liminality that the legality of the naturalizations has been unclear (Abrahamian 2015; Surak 2023). Since exposure, Comoros has stop renewing the passports and disavowed many of the investor citizens, while Vanuatu has lost visa‐free access to the EU.

Yet states do not always give way. Governments can use their regulatory power to stymie the private sector's accumulation of profit, resulting in a *conflictual* relationship or one of outright confrontation. Ministries can delay program implementation, sometimes for years, while the private sector cools its heels, as was the case in Moldova and Saint Lucia. They can be slow in processing applications, hitting private sector profitability, as has happened at various times across the Caribbean. Governments can issue public tenders for new program designs and then revoke them once it receives the policy blueprints, as did Montenegro. They may also turn down the overtures of migration agents who hope to carve out a separate role for themselves in running a program, as did Cyprus, or even cancel a contract with a private agency for such work, as Antigua did in 2014. They may ignore lobbying by agents for program changes attractive to clients—like extending the definition of “dependent children”—as have Turkey and Egypt. In the end, states with their reserves of regulatory capital have the upper hand against the industry, should they choose to wield it, since the private sector has no citizenship to sell without them. In some cases, such pushbacks limit the field's growth, perhaps most obviously when states delay or fail to implement a planned program. But in most instances, it is mainly the profit margins of the private sector that are hit, rather than the field itself.

The type of interface between the public and private described above will condition the amount and kinds of economic capital that programs generate. In many instances, the struggle over a given volume of economic capital comes down to a struggle over what form the money takes, whether as *profits* for the citizenship industry, *kickbacks* for politicians, or *revenue* for the country. Public‐private relationships that are symbiotic or substituting can result in an increase in profits for the private sector and revenue for the country and leave less space for kickbacks. These alliances typically facilitate field growth by generating greater economic capital in a way that strengthens program resilience and legitimacy. Those that are collusive or commandeering will result in private‐sector profits and kickbacks to particular government officials at the expense of country revenue. They also can destabilize the field as illicit practices aimed at personal gain undermine program legitimacy in a field where the acceptability of marketizing a hallowed status is already controversial. Finally, conflictual relationships will see less—if any—profit generated for the private sector and revenue for the country. These, too, will do little to facilitate field growth, but without the more fundamental risks to the field's legitimacy.

Furthermore, close cooperation between governments and the private sector can undermine the state's own symbolic power, which relies on the “misrecognition” of the boundary between states and markets (see Kim 2018). The potential for the boundary between the public and private to be attenuated or erased through collusion or commandeering can carry serious consequences, such as infrastructure projects that never finish or investment or donation money that never enters the country. Even if some form of state cooperation with the private sector is present in—and even necessary for—program operation, the division between the two is one that must be monitored. It is when officials blur the boundaries between public and private for personal benefit, transforming regulatory capital into personal economic benefit, that the greatest risks arise.

### 4.2 Economic Capital in the Competitions Around Regulatory Capital

The rivalry over revenue, however, is possible due only to a state's capacity to issue citizenship, which is itself subject to wider struggles around regulatory capital. It may seem uncontestable that states have sovereign control over whom and how they make a citizen, yet competition over regulatory power has changed both citizenship policies and the field itself. Notably, powerful supranational actors have successfully transformed the naturalization policies of other countries—a remarkable development since citizenship is an exemplary case of a sovereign prerogative. Even international law makes almost no intrusions into this *domaine réservé* of states. The practice of power politics, though, is much different, as actors with superior positions within the hierarchy of states leverage their regulatory power to assert their interests. Yet they do not all or always dominate in the same way. In some cases, the incursions by third powers limit the growth of the CBI market and ultimately threaten the global field, while in others they support the market and regime resilience.

The most prominent contests around regulatory power concern the existence of the programs themselves. Global superpowers and dominant international organizations occupy strong positions within the global field, which supplies leverage over weaker CBI countries. Notably, the European Parliament and European Commission have taken strident stances against the programs, questioning their legitimacy and raising concerns about possibilities for criminal abuse. They have used a variety of tools in these endeavors, from issuing reports and sending fact‐finding missions to CBI countries to formally requesting program revisions or outright closure. Success has been greatest when targeting CBI countries in the accession process. The Commission transforms the promise of future Union membership into regulatory capital by making program closure a de facto requirement to stay on course for joining the EU. This was the strategy it took against Moldova, which shut down its citizenship by investment program in 2020 following threats to revoke EU financial aid and derail accession. Montenegro followed suit by capping its program and then closing it at the beginning of 2023. Under EU pressure, Albania refrained from launching a nascent scheme in 2019, reconfirmed in 2023 (see Surak 2023).

Until recently, the Commission and Parliament have been less successful at pressuring countries already in the club—cases where they lack the possibility of withholding membership and must rely on weaker tools to generate regulatory capital. Cyprus, for example, retained its CBI program in the face of Commission and Parliament pressure until the government froze and then discontinued the scheme following the exposure of high‐level government corruption around it. Malta, too, for several years preserved a CBI offering—and even launched a new one after the first iteration ended—despite continuous Parliament and Commission demands for total closure. However, EU organs have still obtained a degree of regulatory influence. After heated debate in the European Parliament and substantial media exposure, Malta agreed in 2014 to double the application time, increase the investment amount and types, and add requirements for establishing ties to the country. Following similar pressure from 2016 onward, Cyprus began requiring heightened background checks, continued reporting on the investments, and licenses and a code of ethics for service providers. In both cases, the countries made the changes at the EU's behest despite their undesirability in terms of profit‐making, expecting that the sacrifice of potential economic revenue could be converted into regulatory toleration—a conversion of economic loss into potential political gain. Recently, however, the Commission was able to gain the upper hand. On April 29, 2025, the European Court of Justice (ECJ) ruled in its favor—and, notably, against the opinion of the Advocate General—and declared that Malta's citizenship by investment program contravened EU law. The wider impact of the ruling remains to be settled, but it represented an extension of EU power into a new domain, namely naturalization policy, that follows in the long‐established pattern of the ECJ approving the expansion of EU power into areas previously reserved as “competences” of member states (see Horsely 2018).^8^

The European Commission wraps its overt domination in a mantel of symbolic power. “European values are not for sale” is the often‐repeated justification that Commission representatives use when pressuring countries both within and outside the Union. Telling is its recent move against CBI countries. Extraterritorial legal benefits, like visa‐free access, are a key draw for investor citizens. These are secured by treaties with other governments, enabling a CBI country to increase the value of its citizenship by latching onto benefits offered by more powerful or desirable states. However, they also provide a mechanism for influence. In 2023, the Commission proposed an amendment to the EU's visa suspension mechanism that would allow the easy revocation of visa‐free access to the Schengen Area for any country with a CBI program. It validated the change by explaining that “While the Union *respects the right of sovereign countries* to decide on their own naturalisation procedures, visa‐free third countries should be deterred from using visa‐free access to the Union *as a tool for leveraging individual investment* in return for their citizenship” [italics added].^9^ Notably, the Commission gave lip service to doxic principle that sovereign states are able to choose their own naturalization procedures. Yet it also asserted the prerogative to intervene—a regulatory move it justified by declaring that other states should not acquire investments based on legal privileges offered by the EU. Effectively, it drew a line against third countries converting EU regulatory benefits into their own economic capital.

The US, by contrast, has traditionally exercised domination less publicly but more intensively by monitoring and shaping programs through back channels. Leveraging its hegemonic position within the hierarchy of states, it converts its geopolitical strength and geoeconomic necessity into regulatory capital. The most involved branch is the State Department, which views the programs through a security lens, while the Treasury Department has relied on its power over global transactions in US dollars to extract compliance from CBI states regarding financial crime concerns. Remarkably, the State Department has employed its geopolitical power to create a role for itself in the application evaluation process of some countries, which allows the US to transpose its own foreign policy, border control strategies, and security interests onto the naturalization policies of other states. The five Caribbean countries with CBI programs are heavily dependent on the US and on program revenue, which can account for as much as 20% of GDP (Surak 2024). Malta, an EU member state, is less vulnerable but has visa‐free access to the US. Leveraging its regulatory power over these vulnerabilities, the State Department has required the countries to share their lists of CBI applicants before final approval. The US thereby not only gains knowledge of the individuals who may eventually acquire passports, but also the opportunity to reject any applicants it would like to see denied. Washington enhances this regulatory sway by issuing “ban lists” that enumerate nationalities that are disqualified from applying for investor citizenship. When Trump promulgated his first travel ban list in 2017, the Caribbean and Maltese programs fell into line and stopped accepting applications from individuals from those countries. They have subsequently updated the lists of debarred countries to follow changes in US border control and foreign policy. After Russia invaded Ukraine, the US successfully pressed the same countries to stop accepting applications from Russian citizens, with similar results.

Most of these regulatory changes are undesirable from a profit‐making perspective and hinder the strategies CBI states and industry actors use to increase market share and accumulate economic capital. Some countries therefore create carveouts to maneuver into lucrative pockets of demand, such as allowing Iranian citizens who had lived outside Iran for at least 10 years to apply. But in the main, they have fallen into line. Operating in the backroom, the US does not concern itself with shielding its domination through symbolic politics, as does the EU. Still the regulatory capital of a hegemon is substantial, penetrating deep into even the membership decisions of much weaker states in its sphere of influence, as well as hampering economic gains.

However, domination by superpowers is not automatic and CBI countries may allow the regulatory incursions only with reluctance. Cyprus made statements about establishing a separate bureaucratic unit to process applications as requested by the EU but never followed through. Grenada accepted a rush of applications from Russians and Belarusians before implementing the suspension agreed to under US pressure and it continued to process Russian files submitted before the deadline despite US demands to invalidate them. Malta, when pressed to publish the names of investor citizens, released instead those of all naturalizers and listed them in alphabetical order by first name. Dominant regulatory powers do not have unencumbered control, but they meet only weapons of the weak.

On occasion, however, global superpowers have used regulatory influence to push through reforms that, secondarily, facilitate economic gains but have been challenging to implement in a field of actors competing for profit. Two areas in which this has occurred are price competition and vetting coordination. Caribbean CBI governments and the citizenship industry have long complained about the deleterious effects of a “race to the bottom in price” that emerged in the late 2010s as new countries launched programs and lowered minimum investment amounts to increase their market share (Surak 2024). They were also aware of the risks around “venue shopping”—or applicants who are rejected in one country simply taking their file to another—which due diligence companies had long warned about. Both groups had for years discussed possibilities to coordinate policy reforms to solve both issues, but with no action: the competition over economic capital stopped countries from cooperating rather than competing when it came to regulation.

The tragedy‐of‐the‐commons‐style dilemma remained unresolved until dominant third powers intervened to assert their own security interests. In February 2023, The US Treasury Department called a meeting with the Caribbean countries and compelled them to adopt a set of policy reforms, known as the “Six Principles,” that required countries to carry out enhanced background checks and regular audits, as well as share information about denials to prevent venue shopping.^10^ The countries, which are heavily dependent on the easy‐clearing of US correspondent banks for their economic survival, implemented the reforms and transformed their naturalization systems to meet US demands. A year later, the European Commission obliged the Caribbean CBI countries to sign a Memorandum of Agreement that reinforced the Six Principles and added a requirement to raise the donation and investment floor to a minimum $200,000.^11^ The governments, concerned that they might lose visa‐free access to the Schengen Zone of the European Union, again complied. The move drew a line under the price competition that led to falling investment amounts over the prior decade while serving the EU's security concerns in reducing the number of people moving through the programs.

Even when coerced to comply with the interests of powerful external actors, CBI countries recast their submission into an expression of tacit countenance from dominant players to shore up legitimacy, which remains unstable within the global field. Indeed, several have converted regulatory pressure into profitable selling points. After conceding to changes called for the European Parliament, Maltese government officials began pitching their program as the only one “endorsed” by the European Union as they attempted to transform the regulatory loss into economic gains (Surak 2023). Officials in Saint Kitts have adopted a similar strategy. When the European Commission began signaling a desire for Caribbean countries to raise their minimum investment amount, the government quickly doubled its price in July 2023, nearly a year ahead of the expected deadline. The immediate result for Saint Kitts was a substantial decline in applications received, with reportedly less than a dozen approved in the following months. However, officials saw the move as a long‐term strategy to stay ahead of the game, court the good will of the EU, and claim the pole position among competitors. In the following months at CBI events such as one in Dubai in April 2024, the prime minister touted the country's leadership in the field: “We are setting the standard of good governance… There is a European proposal for suspending visa‐free [access]. We have already been making all the significant changes that they want. We are ahead of the game. Now in hindsight, we can understand why. We are not reactive but proactive.” Locating the country at the front of the pack, he listed other recent program changes—all concessions made to third powers—but reframed them as their own, declaring that “Saint Kitts will continue to be innovative.” In the face of regulatory incursions and losses, countries can attempt to flip their weakness into market strength by leveraging the connection to dominant powers for the fortification of legitimacy and, simultaneously, symbolic power within the field.

## 5 Conclusion

A global field approach supplies the tools for unpacking how migration regimes are shaped by states and migration industries that vie for economic benefits within a complex, multilevel regulatory landscape. Reframing migration industries and multilevel migration governance as part of the same arena in which unequal actors struggle over regulatory and economic power reveals how the power‐plays over economic capital intersect with those over regulatory capital and the effects they have on the growth or decline of a migration regime. It also facilitates the analysis of the fundamental misrecognitions—manipulations of symbolic power—that undergird legitimacy and enable the field to exist in the first place. The result is a more comprehensive understanding of the ways in which migration regimes are shaped through the creation, accumulation, leveraging, conversion, and loss of both economic and regulatory capital.

The case study revealed several benefits of a global field approach. Focusing first on economic capital competition, it showed how regulatory powers fundamentally condition the struggle for profit: country borders establish the information asymmetries that generate profit and the legal limits that produce protection for the migration industry's extended supply chain. Extending the literature on migration industries, the analysis identified key forms of public‐private relationships at play based on the exchanges between economic and regulatory capital, and it used these to show how the different forms can facilitate or stymie the market's growth. CBI countries actively engage with players in the migration industry as they lobby state actors with the promise of profit to convert the country's regulatory power into economic revenue. Forming public‐private relationships that are *symbiotic* or *substituting* can enhance market's growth and resilience, increasing the overall volume of economic capital, both as profits for the private sector and revenue for the state. Those that are *collusive* or *commandeering*, however, transform potential state revenue into private sector profit and kickbacks for politicians and erode resilience and legitimacy. Finally, *conflictual* relationships stymie the overall growth of economic capital in the field, whether as profits, revenue, or kickbacks. Furthermore, the collaborations contain risks, for narrowing the distance between the public and private can undermine the state's own symbolic power.

Turning next to regulatory competition, the case study revealed that states are not always the “central bank of symbolic capital” (cf. Bourdieu 1997, 240) nor do they, without question, hold “the monopoly of legitimate symbolic violence” (cf. Bourdieu 1997, 186). Rather, they too exist in the uneven global playing fields. Attending to the dynamics of capital accumulation and conversion exposes how and why external actors can manipulate regulatory capital to gain influence over another state's citizenship policies, even without the formal legal backing to do so.

Extending the literature on multilevel migration management, the analysis showed how influential external powers like third states and distant supranational organizations can shape outcomes not merely through issue linkage but by drawing on their superior positions within the hierarchy of states to advance their own interests. The upshot enables third powers to successfully coerce other countries to end programs or change their design, and even intervene in naturalization processes to gain a say over who can apply and who must be refused. Such domination may reduce the volume of economic capital that a CBI country accrues from a program, but as subordinated actors among the political powers in the wider global field, they comply, if sometimes with reluctance. However, not all interventions by dominant powers reduce the space of play. Indeed, some incursions strengthen its operation and the possibilities to accumulate economic capital, impacting the operation of the migration industry. Examples include coordination around minimum prices and background checks. In these cases, third powers have successfully brought countries locked in economic competition to the table to establish regulatory minimums that boost the resilience and growth of citizenship by investment. To date, it has been backroom domination that has, in end effect, enhanced the possibilities for market expansion. By contrast, overt domination, wielded with larger reserves of symbolic capital, has stymied it.

Finally, the analysis exposed the field's doxa, including that states possess a monopoly on the legitimate authority over citizenship. The global field approach revealed this as not only an arbitrary truth, but also one that dominant powers like the EU may leverage to cloak their domination with symbolic power. Indeed, dominant powers dominate differently in their incursions into state sovereignty over citizenship. The US has exerted influence primarily behind the scenes, without the need for recourse to symbolic violence to cover its maneuvers. Furthermore, its backchannel power has been a more penetrating intervention into sovereignty as the US has obtained direct control over who can naturalize in a country. The EU, by contrast, has wielded its regulatory power overtly, if often bluntly aimed at shutting down programs, and it has swathed its overt domination in symbolic power by questioning the legitimacy of citizenship by investment.

Though the case study discussed here focused solely on citizenship by investment, a global field approach can be applied beyond it. Refugees, for example, are frequently deemed an exemplary case of political migrants. However, a growing body of work has drawn attention to how states monetize refugee flows (Andersson 2014; FitzGerald 2019; Adamson and Tsourapas 2019; Freier et al. 2021). A field analysis of refugee politics could illuminate the complex scrambles between host or transit countries, like Turkey or Jordan, that seek to convert regulatory capital into economic revenue, and how dominant actors, like the EU, US, and Australia, attempt to externalize border control by transforming economic funding into regulatory influence in third countries. A global field approach would supply tools for examining how the politicized contestations over who counts as a refugee can itself be a source of capital. As a framework that casts state power as variable and states as not always operating effectively or with internal coherence, it offers tools for comparing across cases in which state infrastructural power is weak or challenged and IGOs and NGOs play crucial governance roles. It could also be rallied to identify when and how migration diplomacy utilizes the monetization of refugee populations for leverage over dimensions of foreign relations. In sum, a global fields approach could offer leverage for a more thorough dissection of the ways that profit and politics operate in establishing and undergirding migration regimes.

## Ethics Statement

Ethical approval was obtained from the London School of Economics.

## Conflicts of Interest

The author declares no conflicts of interest.

## Data Availability

The author has nothing to report.

## Endnotes

^1^
Bourdieu's “global economic field” is similar in scope and in the key actors included—namely states, corporations, and international organizations—but rather than focusing on political competition, he uses the concept to analyze economic globalization (Bourdieu 2005).

^2^
Note that although Go (2008) introduces his approach with the term “global political field,” he shortens it to “global field” in his analysis.

^3^
The key exception under international law is that a state is obliged to allow its citizens to enter. (However, the converse, allowing citizens to leave, is a sovereign prerogative.)

^4^
Note that although “conversion” is the commonly used verb, particularly in Bourdieusian approaches to field analysis, it does not always imply that one form of capital disappears while another is conjured into existence (see Bourdieu 2002). Wealth may be expended when acquiring an elite education, for example, but—conversely—cultural capital is not erased when a qualification from an elite academic institution is used to obtain a high‐paying job. “Leveraging” is perhaps a more apt descriptor in such cases.

^5^
EU citizenship is derivative of citizenship in a member state and not issued by the EU.

^6^
The weaker end of the capital flows and competitions includes locals in the country offering citizenship by investment and the wealthy naturalizers themselves, two groups bracketed here for the sake of concision.

^7^
The wider ecology of the citizenship industry extends beyond the firms involved with submitting applications to include several additional types of private sector businesses that are less relevant here. Real estate developers may build properties specifically aimed at the citizenship by investment market or advertise their offerings for it. Financial service firms may assist individuals with moving money across borders, funding their investment, avoiding currency controls, and even producing funds that tick the right boxes of “cleanliness” to be moved internationally and accepted by a country. These businesses may be linked through “tie ups” or reciprocal relationships to migration agents, and some agents may have subdivisions that address these attendant services as well. In addition, international due diligence companies can be appointed by both governments and migration service providers to run background checks on and vet applicants.

^8^
See Surak (2023) for a discussion of the powerplays and stakes in the proceedings which continued over several years.

^9^
European Commission, “Proposal for a Regulation of the European Parliament and of the Council Amending Regulation (EU) 2018/1806 as Regards the Revision of the Suspension Mechanism.” COM(2023) 642 final. 2023/0371 (COD).

^10^
The other principles include the mandatory interviews of applicants, retrieving revoked passports, and the suspension of applications from Russia and Belarus.

^11^
It included a reaffirmation of the Six Principles and the agreement to establish a regional regulatory authority, as well as joint training, increased information sharing and reporting, and the regulation of agents and marketing.
